# 6,7-Di­bromo-2-methyl-3-phenyl­quinoxaline

**DOI:** 10.1107/S2414314626010278

**Published:** 2026-10-09

**Authors:** Avion T. Valentin, Guy Crundwell, Gary S. Nichol

**Affiliations:** a Department of Chemistry & Biochemistry Central Connecticut State University, 1619 Stanley Street New Britain CT 06053 USA; b The University of Edinburgh School of Chemistry Joseph Black Building David Brewster Road Edinburgh EH9 3FJ United Kingdom; Purdue University, USA

**Keywords:** crystal structure, quinoxaline

## Abstract

The title com­pound, C_15_H_10_Br_2_N_2_, was synthesized using the acid catalyst NH_4_HF_2_ in a water/methanol solution. There are two mol­ecules per asymmetric unit where the quinoxaline moieties make dihedral angles of 48.05 (4) and 40.83 (5)° with respect to their phenyl rings. In the extended structure, mol­ecules form layers stacked along (001) that are held together by hy­dro­gen and halogen bonds.

## Abstract 2

The title com­pound was synthesized using the acid catalyst NH_4_HF_2_ in a water/methanol solution.

## Graphical abstract

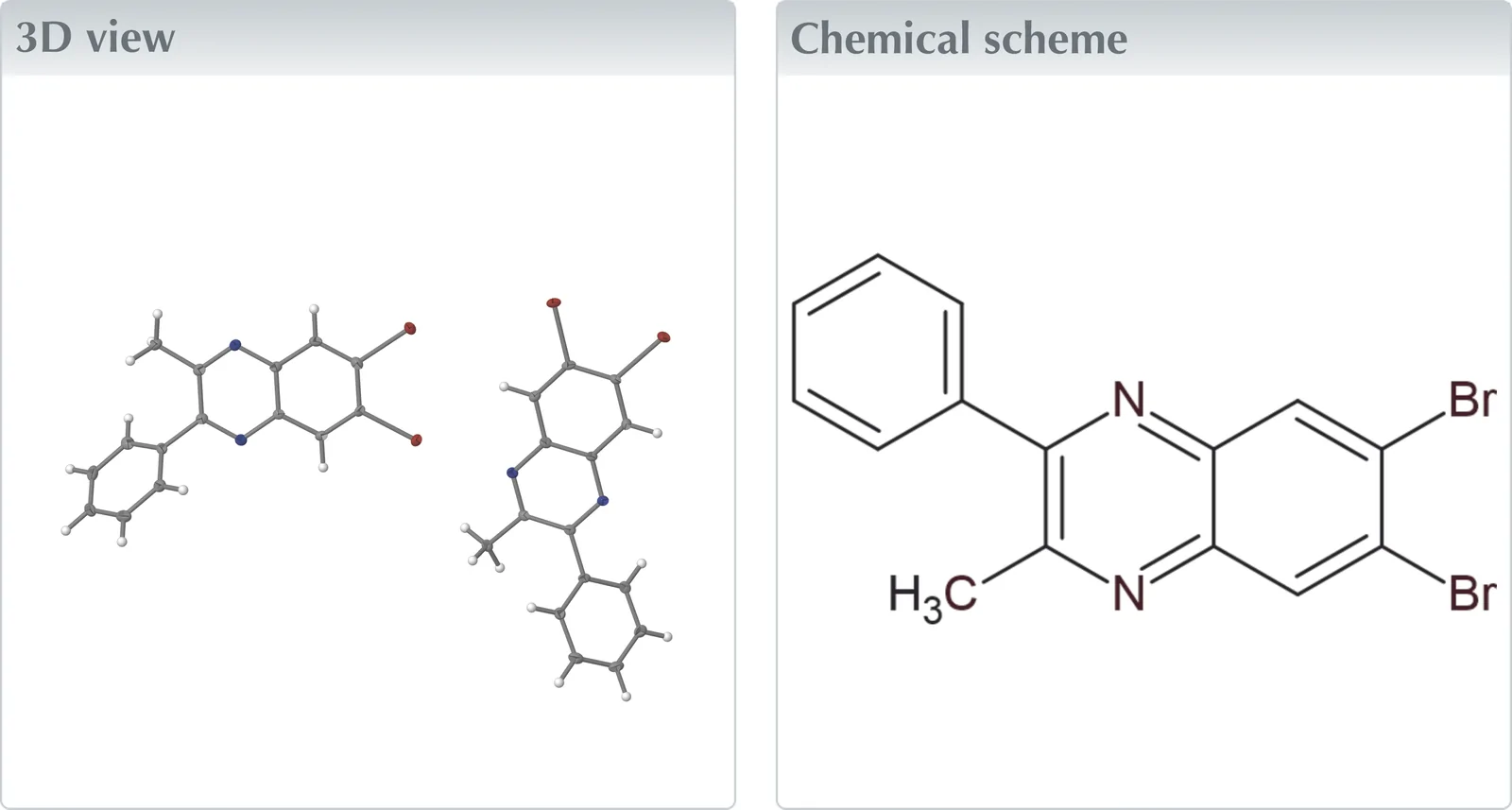

## Structure description

In general, quinoxalines are of inter­est as potential anti­bacterial (Parhi *et al.*, 2013 ▸), anti­microbial (Singh *et al.*, 2010 ▸), and anti­fungal medicines (Tang *et al.*, 2022 ▸); therefore, exploring their synthesis and structures is of inter­est. The title com­pound was synthesized *via* a condensation reaction of a di­amine and diketone using a water/methanol solution containing a catalytic amout of NH_4_HF_2_ catalyst (Lassagne *et al.*, 2015 ▸).

**Figure 1:**
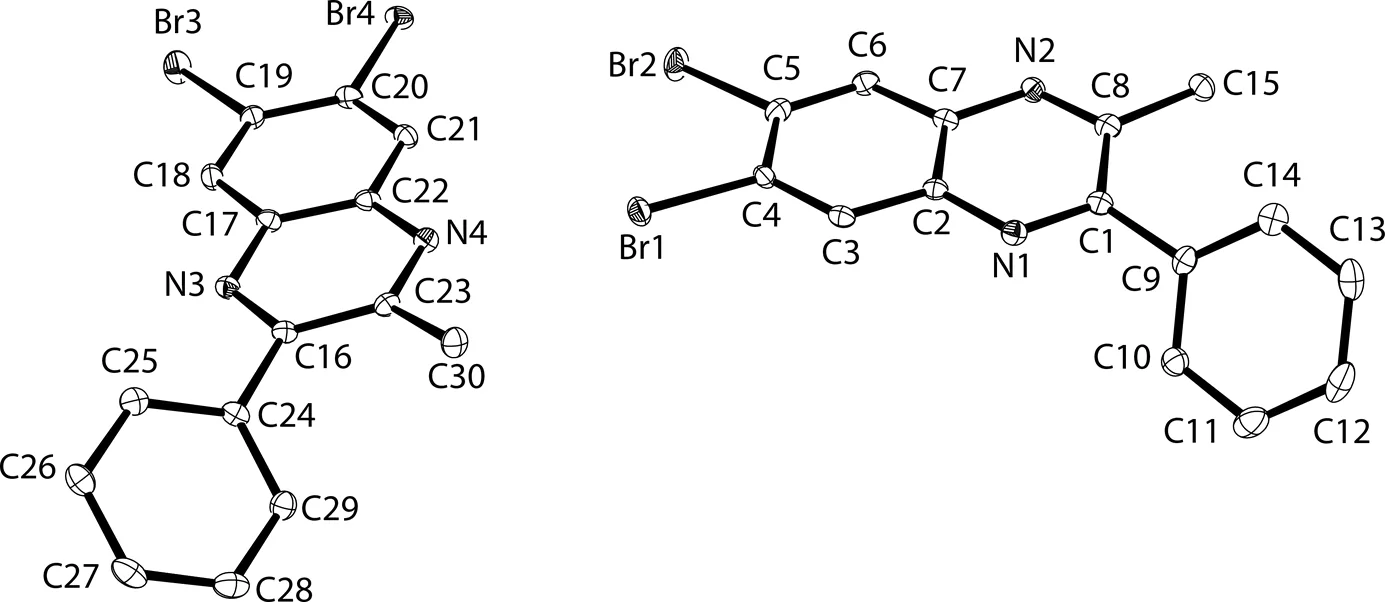
A view of the molecules of the title com­pound, with displacement ellipsoids drawn at the 50% probability level and H atoms omitted for clarity.

**Figure 2:**
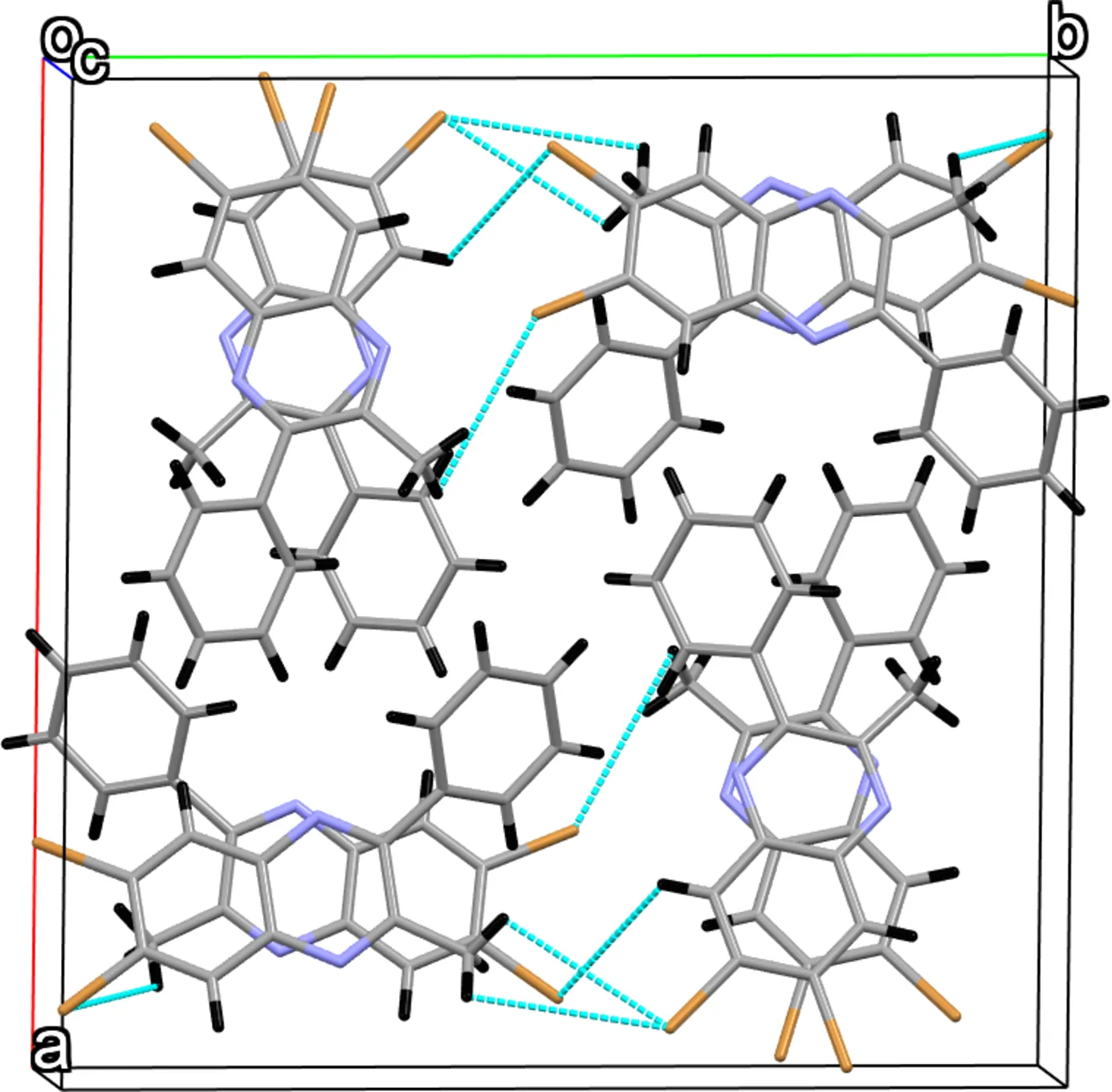
A view of unit-cell packing along [001], with some of the C—H⋯Br hy­dro­gen-bonding inter­actions shown.

**Figure 3:**
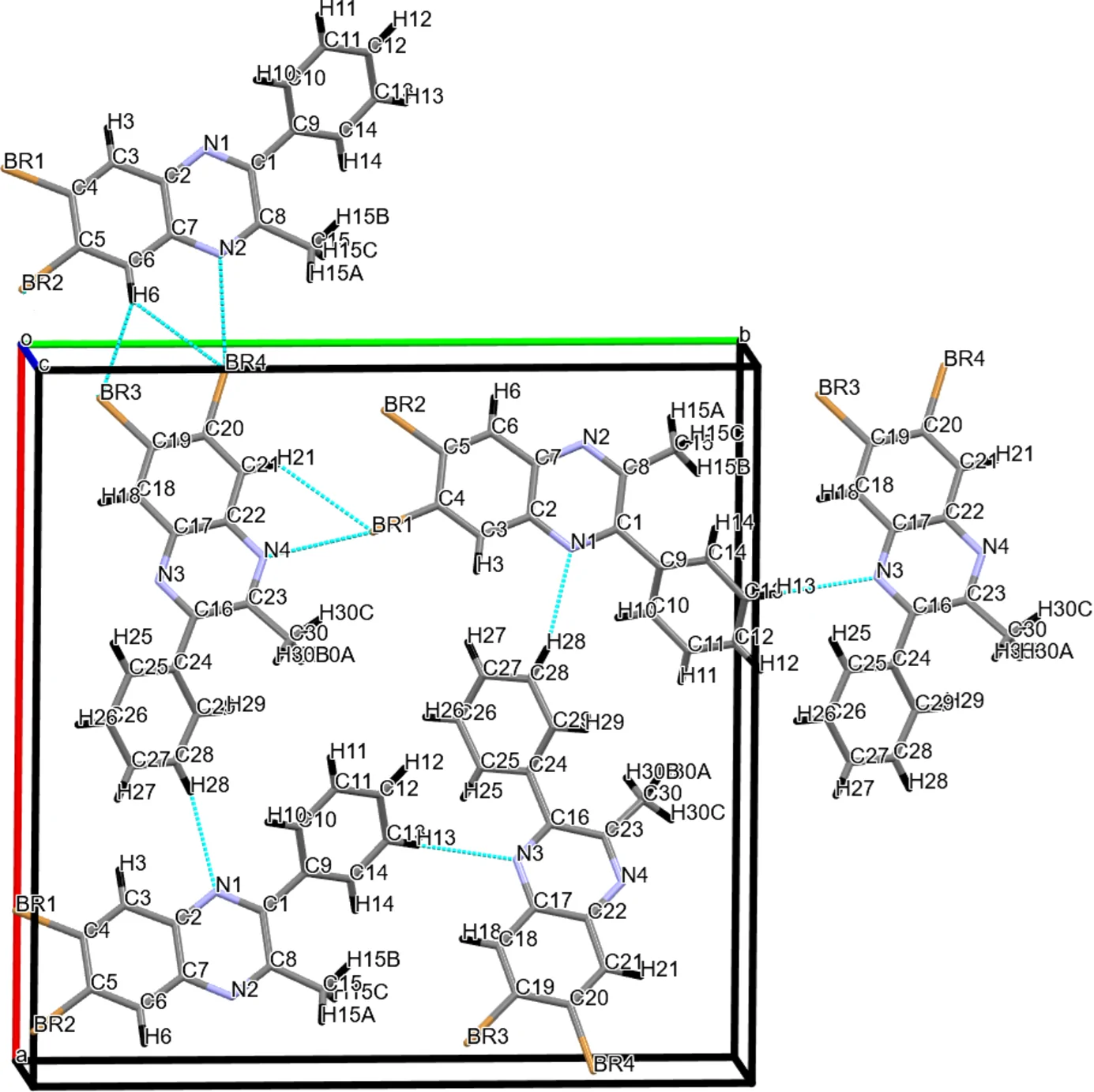
A view of the inter­molecular inter­actions (halogen and hy­dro­gen bonds) in layers along the (001) direction.

There are two mol­ecules per asymmetric unit (Fig. 1 ▸) where the quinoxaline moieties make dihedral angles of 48.05 (4) and 40.83 (5)° with respect to their phenyl rings. Mol­ecules pack in a herringbone pattern where quinoxalines form planes (Fig. 2 ▸). There are two types of inter­molecular inter­actions – halogen bonds and hy­dro­gen bonds. Fig. 3 ▸ shows the halogen and hy­dro­gen bonds between mol­ecules that lie normal to the (001) direction of the unit cell. The hy­dro­gen bonds in this layer are between C6—H6⋯Br3^i^, C6—H6⋯Br4^i^, C13—H13⋯N3^iii^, C21—H21⋯Br1, and C28—H28⋯N1^v^ (see Table 1 ▸ for symmetry codes and metrics). The halogen bonds involve Br1⋯N4 [3.0230 (19) Å, with C1—Br1⋯N4 = 171.29 (8)°] and Br4⋯N2^vi^ [3.2270 (18) Å, with C4—Br4⋯N2^vi^ = 160.96 (8)°; symmetry code: (vi) −*x*, *y* − 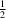, −*z* + 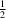]. The hy­dro­gen bonds in Table 2 ▸ that have not been specifically mentioned involve methyl groups and connect mol­ecules between layers.

## Synthesis and crystallization

In a 50 ml Erlenmeyer flask, 1.48 g of 1-phenyl­propane-1,2-dione (10.0 mmol) was addded to 2 ml of H_2_O and 20 ml of a 2.5 × 10^−3^ *M* methanol solution of NH_4_HF_2_ (Lassagne *et al.*, 2015 ▸). To this mixture, 2.66 g of 4,5-di­bromo-1,2-phenyl­enedi­amine (10.0 mmol) was added. The solution was stirred for 24 h, during which time the product precipitated as a pink–white product. 3.48 g of crude product was isolated and recrysallized from methanol solution, yielding 1.60 g of white purified powder (42%). The yield was low due to loss of material during recrystallization. Crystals for diffraction study were isolated from the slow evaporation of a CH_2_Cl_2_ solution (m.p. 416 K). ^1^H NMR (CDCl_3_, 300 MHz): δ 2.72 (*s*, 3H), 7.50 (*m*, 3H), 7.61 (*m*, 2H), 8.30 (*s*, 1H), 8.35 (*s*, 1H); ^13^C NMR (CDCl_3_, 300 MHz): δ 24.54, 125.65, 126.20, 128.65, 128.90, 129.48, 132.42, 133.17, 138.18, 140.21, 140.34, 154.14, 155.99.

## Refinement

Crystal data, data collection and structure refinement details are summarized in Table 2 ▸. H atoms on *sp*^2^ and *sp*^3^ C atoms were placed at calculated positions with C—H distances of 0.95 and 0.98 Å, respectively, and were included in the refinement in riding motion approximation with *U*_iso_(H) = 1.2*U*_eq_ or 1.5*U*_eq_ of the carrier atom, respectively.

## Supplementary Material

Crystal structure: contains datablock(s) I. DOI: 10.1107/S2414314626010278/zl4104sup1.cif

Structure factors: contains datablock(s) I. DOI: 10.1107/S2414314626010278/zl4104Isup2.hkl

Supporting information file. DOI: 10.1107/S2414314626010278/zl4104Isup3.cml

CCDC reference: 2593016

Additional supporting information:  crystallographic information; 3D view; checkCIF report

Additional supporting information:  crystallographic information; 3D view; checkCIF report

## Figures and Tables

**Table 1:** Hydrogen-bond geometry (Å, °)

*D*—H⋯*A*	*D*—H	H⋯*A*	*D*⋯*A*	*D*—H⋯*A*
C6—H6⋯Br3^i^	0.95	2.91	3.784 (2)	154
C6—H6⋯Br4^i^	0.95	3.09	3.834 (2)	136
C15—H15*B*⋯Br1^ii^	0.98	3.13	4.044 (2)	155
C21—H21⋯Br1	0.95	3.05	3.741 (2)	131
C13—H13⋯N3^iii^	0.95	2.62	3.495 (3)	152
C28—H28⋯N1^v^	0.95	2.68	3.568 (3)	156

Symmetry codes: (i) 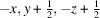; (ii) 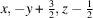; (iii) 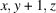; (iv) 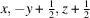; (v) 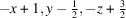.

**Table 2:** Experimental details

Crystal data	
Chemical formula	C_15_H_10_Br_2_N_2_
*M*_r_	378.07
Crystal system, space group	Monoclinic, *P*2_1_/*c*
Temperature (K)	100
*a*, *b*, *c* (Å)	19.0464 (3), 18.6850 (4), 7.5964 (2)
β (°)	97.495 (2)
*V* (Å^3^)	2680.32 (10)
*Z*	8
Radiation type	Mo *K*α
μ (mm^−1^)	6.03
Crystal size (mm)	0.28 × 0.21 × 0.12 × 0.09 (radius)

Data collection	
Diffractometer	Rigaku XtaLAB Synergy Single source diffractometer with a HyPix-Arc 100 detector
Absorption correction	For a sphere (*CrysAlis PRO*; Rigaku OD, 2024 ▸)
*T*_min_, *T*_max_	0.420, 0.436
No. of measured, independent and observed [*I* > 2σ(*I*)] reflections	44577, 9561, 7295
*R*_int_	0.047
(sin θ/λ)_max_ (Å^−1^)	0.784

Refinement	
*R*[*F*^2^ > 2σ(*F*^2^)], *wR*(*F*^2^), *S*	0.043, 0.065, 1.05
No. of reflections	9561
No. of parameters	345
H-atom treatment	H-atom parameters constrained
Δρ_max_, Δρ_min_ (e Å^−3^)	0.68, −0.54

Computer programs: *CrysAlis PRO* (Rigaku OD, 2024 ▸), *SHELXT* (Sheldrick, 2015*a* ▸), *SHELXL2025* (Sheldrick, 2015*b* ▸), *ORTEP-3* (Farrugia, 2012 ▸), *Mercury* (Macrae *et al.*, 2020 ▸), and *ShelXle* (Hübschle *et al.*, 2011 ▸).

## full crystallographic data

### 6,7-Dibromo-2-methyl-3-phenylquinoxaline Crystal data

**Table:**

C_15_H_10_Br_2_N_2_	*D*_x_ = 1.874 Mg m^−^^3^
*M**_r_* = 378.07	Melting point: 416 K
Monoclinic, *P*2_1_/*c*	Mo *K*α radiation, λ = 0.71073 Å
*a* = 19.0464 (3) Å	Cell parameters from 15741 reflections
*b* = 18.6850 (4) Å	θ = 2.9–32.9°
*c* = 7.5964 (2) Å	µ = 6.03 mm^−^^1^
β = 97.495 (2)°	*T* = 100 K
*V* = 2680.32 (10) Å^3^	Block, colorless
*Z* = 8	0.28 × 0.21 × 0.12 × 0.09 (radius) mm
*F*(000) = 1472	

### 6,7-Dibromo-2-methyl-3-phenylquinoxaline Data collection

**Table:**

Rigaku XtaLAB Synergy Single source diffractometer with a HyPix-Arc 100 detector	9561 independent reflections
Radiation source: fine-focus sealed X-ray tube, Enhance (Mo) X-ray Source	7295 reflections with *I* > 2σ(*I*)
Graphite monochromator	*R*_int_ = 0.047
Detector resolution: 10.0000 pixels mm^-1^	θ_max_ = 33.9°, θ_min_ = 2.9°
ω scans	*h* = −29→27
Absorption correction: for a sphere (CrysAlis PRO; Rigaku OD, 2024)	*k* = −29→24
*T*_min_ = 0.420, *T*_max_ = 0.436	*l* = −11→11
44577 measured reflections	

### 6,7-Dibromo-2-methyl-3-phenylquinoxaline Refinement

**Table:**

Refinement on *F*^2^	Primary atom site location: SHELXT (Sheldrick, 2015*a*)
Least-squares matrix: full	Hydrogen site location: inferred from neighbouring sites
*R*[*F*^2^ > 2σ(*F*^2^)] = 0.043	H-atom parameters constrained
*wR*(*F*^2^) = 0.065	*w* = 1/[σ^2^(*F*_o_^2^) + (0.0185*P*)^2^ + 2.9293*P*] where *P* = (*F*_o_^2^ + 2*F*_c_^2^)/3
*S* = 1.05	(Δ/σ)_max_ = 0.001
9561 reflections	Δρ_max_ = 0.68 e Å^−^^3^
345 parameters	Δρ_min_ = −0.54 e Å^−^^3^
0 restraints	

### 6,7-Dibromo-2-methyl-3-phenylquinoxaline Special details

**Table:**

Geometry. All esds (except the esd in the dihedral angle between two l.s. planes) are estimated using the full covariance matrix. The cell esds are taken into account individually in the estimation of esds in distances, angles and torsion angles; correlations between esds in cell parameters are only used when they are defined by crystal symmetry. An approximate (isotropic) treatment of cell esds is used for estimating esds involving l.s. planes.
Refinement. 1. Fixed Uiso At 1.2 times of: All C(H) groups At 1.5 times of: All C(H,H,H) groups 2.a Aromatic/amide H refined with riding coordinates: C3(H3), C6(H6), C10(H10), C11(H11), C12(H12), C13(H13), C14(H14), C18(H18), C21(H21), C25(H25), C26(H26), C27(H27), C28(H28), C29(H29) 2.b Idealised Me refined as rotating group: C15(H15a,H15b,H15c), C30(H30a,H30b,H30c)

### 6,7-Dibromo-2-methyl-3-phenylquinoxaline Fractional atomic coordinates and isotropic or equivalent isotropic displacement parameters (Å^2^)

**Table:**

	*x*	*y*	*z*	*U*_iso_*/*U*_eq_	
Br1	0.24220 (2)	0.47648 (2)	0.59870 (3)	0.01589 (5)	
Br2	0.08173 (2)	0.49698 (2)	0.36507 (3)	0.02061 (6)	
N1	0.26864 (10)	0.75369 (10)	0.5630 (2)	0.0127 (4)	
N2	0.12777 (10)	0.77270 (10)	0.4032 (2)	0.0130 (4)	
C1	0.24242 (11)	0.81804 (12)	0.5236 (3)	0.0118 (4)	
C2	0.22464 (11)	0.69655 (12)	0.5254 (3)	0.0108 (4)	
C3	0.25015 (11)	0.62685 (12)	0.5712 (3)	0.0123 (4)	
H3	0.296657	0.620597	0.631582	0.015*	
C4	0.20793 (11)	0.56870 (12)	0.5287 (3)	0.0120 (4)	
C5	0.13873 (12)	0.57774 (13)	0.4358 (3)	0.0145 (4)	
C6	0.11233 (11)	0.64483 (12)	0.3933 (3)	0.0137 (4)	
H6	0.065779	0.650351	0.332637	0.016*	
C7	0.15481 (11)	0.70558 (12)	0.4404 (3)	0.0120 (4)	
C8	0.16899 (11)	0.82787 (12)	0.4477 (3)	0.0129 (4)	
C9	0.29498 (11)	0.87743 (12)	0.5513 (3)	0.0130 (4)	
C10	0.36089 (12)	0.86606 (13)	0.4938 (3)	0.0156 (5)	
H10	0.369780	0.822506	0.436123	0.019*	
C11	0.41331 (13)	0.91774 (14)	0.5203 (3)	0.0218 (5)	
H11	0.457800	0.909752	0.479885	0.026*	
C12	0.40080 (13)	0.98142 (13)	0.6062 (3)	0.0225 (5)	
H12	0.436954	1.016625	0.625970	0.027*	
C13	0.33547 (13)	0.99332 (13)	0.6627 (3)	0.0200 (5)	
H13	0.326855	1.036866	0.720752	0.024*	
C14	0.28258 (13)	0.94190 (13)	0.6348 (3)	0.0161 (5)	
H14	0.237760	0.950631	0.672709	0.019*	
C15	0.13594 (12)	0.90040 (12)	0.4132 (3)	0.0157 (5)	
H15A	0.095392	0.896590	0.319925	0.024*	
H15B	0.170950	0.933389	0.374788	0.024*	
H15C	0.119921	0.918504	0.522263	0.024*	
Br3	0.06220 (2)	0.10061 (2)	0.38293 (3)	0.02061 (6)	
Br4	0.02408 (2)	0.27571 (2)	0.34126 (3)	0.01466 (5)	
N3	0.30515 (9)	0.17557 (10)	0.7234 (2)	0.0121 (4)	
N4	0.27362 (9)	0.32225 (10)	0.7003 (2)	0.0120 (4)	
C16	0.35225 (11)	0.22414 (12)	0.7863 (3)	0.0103 (4)	
C17	0.24113 (11)	0.19876 (12)	0.6434 (3)	0.0115 (4)	
C18	0.19038 (11)	0.14799 (12)	0.5711 (3)	0.0137 (4)	
H18	0.200116	0.098282	0.584403	0.016*	
C19	0.12717 (11)	0.17060 (12)	0.4820 (3)	0.0127 (4)	
C20	0.11180 (11)	0.24510 (13)	0.4634 (3)	0.0129 (4)	
C21	0.16011 (11)	0.29493 (12)	0.5334 (3)	0.0123 (4)	
H21	0.149460	0.344479	0.520711	0.015*	
C22	0.22615 (11)	0.27262 (12)	0.6252 (3)	0.0114 (4)	
C23	0.33522 (11)	0.29920 (12)	0.7821 (3)	0.0111 (4)	
C24	0.42317 (11)	0.19515 (12)	0.8601 (3)	0.0114 (4)	
C25	0.42573 (12)	0.13067 (12)	0.9540 (3)	0.0132 (4)	
H25	0.382968	0.107428	0.972814	0.016*	
C26	0.49055 (12)	0.10032 (13)	1.0202 (3)	0.0155 (5)	
H26	0.491937	0.056718	1.084880	0.019*	
C27	0.55319 (12)	0.13373 (13)	0.9918 (3)	0.0181 (5)	
H27	0.597436	0.113425	1.038665	0.022*	
C28	0.55113 (12)	0.19686 (13)	0.8948 (3)	0.0172 (5)	
H28	0.594027	0.218994	0.872762	0.021*	
C29	0.48638 (11)	0.22790 (12)	0.8297 (3)	0.0138 (4)	
H29	0.485254	0.271393	0.764633	0.017*	
C30	0.38241 (12)	0.35517 (12)	0.8767 (3)	0.0141 (4)	
H30A	0.415288	0.372920	0.797704	0.021*	
H30B	0.409292	0.334255	0.983132	0.021*	
H30C	0.353484	0.394892	0.911121	0.021*	

### 6,7-Dibromo-2-methyl-3-phenylquinoxaline Atomic displacement parameters (Å^2^)

**Table:**

	*U*^11^	*U*^22^	*U*^33^	*U*^12^	*U*^13^	*U*^23^
Br1	0.01469 (10)	0.01104 (11)	0.02134 (12)	0.00220 (8)	0.00010 (8)	0.00060 (9)
Br2	0.01600 (11)	0.01336 (11)	0.03052 (14)	−0.00353 (9)	−0.00431 (9)	0.00033 (10)
N1	0.0122 (9)	0.0133 (9)	0.0125 (9)	−0.0003 (7)	0.0011 (7)	−0.0007 (7)
N2	0.0124 (9)	0.0125 (9)	0.0139 (9)	0.0013 (7)	0.0008 (7)	0.0013 (7)
C1	0.0123 (10)	0.0140 (11)	0.0091 (10)	0.0009 (8)	0.0015 (8)	−0.0006 (8)
C2	0.0107 (9)	0.0133 (11)	0.0087 (10)	0.0006 (8)	0.0022 (7)	0.0001 (8)
C3	0.0093 (10)	0.0146 (11)	0.0130 (11)	0.0022 (8)	0.0013 (8)	0.0003 (8)
C4	0.0125 (10)	0.0105 (11)	0.0134 (11)	0.0031 (8)	0.0032 (8)	0.0009 (8)
C5	0.0139 (10)	0.0150 (11)	0.0147 (11)	−0.0033 (8)	0.0025 (8)	−0.0007 (9)
C6	0.0101 (10)	0.0167 (12)	0.0138 (11)	−0.0008 (8)	−0.0007 (8)	0.0004 (9)
C7	0.0110 (10)	0.0140 (11)	0.0112 (10)	0.0016 (8)	0.0018 (8)	−0.0003 (8)
C8	0.0143 (10)	0.0145 (11)	0.0100 (10)	0.0010 (8)	0.0022 (8)	0.0016 (8)
C9	0.0140 (10)	0.0124 (11)	0.0118 (11)	−0.0018 (8)	−0.0016 (8)	0.0029 (8)
C10	0.0155 (11)	0.0142 (12)	0.0168 (12)	0.0009 (9)	0.0013 (9)	0.0021 (9)
C11	0.0141 (11)	0.0240 (14)	0.0265 (14)	−0.0015 (10)	−0.0010 (9)	0.0084 (10)
C12	0.0206 (12)	0.0162 (12)	0.0276 (14)	−0.0068 (9)	−0.0081 (10)	0.0085 (10)
C13	0.0276 (13)	0.0118 (12)	0.0186 (12)	0.0006 (10)	−0.0049 (10)	0.0007 (9)
C14	0.0186 (11)	0.0173 (12)	0.0117 (11)	−0.0007 (9)	−0.0008 (9)	0.0009 (9)
C15	0.0136 (10)	0.0135 (11)	0.0196 (12)	0.0031 (9)	0.0001 (8)	0.0025 (9)
Br3	0.01343 (11)	0.01599 (12)	0.02968 (14)	−0.00320 (9)	−0.00745 (9)	−0.00083 (10)
Br4	0.00940 (9)	0.01911 (12)	0.01489 (11)	0.00257 (8)	−0.00061 (7)	0.00171 (9)
N3	0.0095 (8)	0.0123 (9)	0.0144 (9)	0.0008 (7)	0.0009 (7)	0.0002 (7)
N4	0.0118 (8)	0.0124 (9)	0.0115 (9)	−0.0006 (7)	0.0006 (7)	−0.0003 (7)
C16	0.0103 (9)	0.0123 (11)	0.0086 (10)	−0.0001 (8)	0.0014 (7)	−0.0022 (8)
C17	0.0093 (9)	0.0130 (11)	0.0118 (10)	0.0001 (8)	−0.0001 (8)	−0.0011 (8)
C18	0.0129 (10)	0.0097 (11)	0.0181 (11)	0.0004 (8)	0.0005 (8)	0.0005 (8)
C19	0.0092 (9)	0.0125 (11)	0.0157 (11)	−0.0023 (8)	−0.0007 (8)	−0.0007 (8)
C20	0.0094 (9)	0.0182 (12)	0.0106 (11)	0.0021 (8)	−0.0009 (8)	0.0026 (8)
C21	0.0126 (10)	0.0126 (11)	0.0116 (10)	0.0027 (8)	0.0018 (8)	0.0012 (8)
C22	0.0108 (9)	0.0135 (11)	0.0105 (10)	−0.0001 (8)	0.0033 (8)	−0.0021 (8)
C23	0.0118 (10)	0.0130 (11)	0.0086 (10)	−0.0018 (8)	0.0020 (8)	−0.0004 (8)
C24	0.0098 (9)	0.0140 (11)	0.0097 (10)	0.0013 (8)	−0.0011 (7)	−0.0027 (8)
C25	0.0145 (10)	0.0133 (11)	0.0119 (11)	−0.0014 (8)	0.0019 (8)	−0.0041 (8)
C26	0.0171 (11)	0.0143 (11)	0.0145 (11)	0.0034 (9)	−0.0001 (8)	−0.0024 (9)
C27	0.0129 (11)	0.0204 (13)	0.0199 (12)	0.0051 (9)	−0.0023 (9)	−0.0043 (10)
C28	0.0117 (10)	0.0217 (13)	0.0182 (12)	−0.0011 (9)	0.0021 (9)	−0.0051 (9)
C29	0.0148 (10)	0.0128 (11)	0.0136 (11)	−0.0014 (8)	0.0008 (8)	−0.0003 (8)
C30	0.0153 (10)	0.0123 (11)	0.0141 (11)	−0.0022 (8)	−0.0007 (8)	−0.0022 (8)

### 6,7-Dibromo-2-methyl-3-phenylquinoxaline Geometric parameters (Å, º)

**Table:**

Br1—C4	1.894 (2)	Br3—C19	1.888 (2)
Br2—C5	1.895 (2)	Br4—C20	1.891 (2)
N1—C1	1.321 (3)	N3—C16	1.321 (3)
N1—C2	1.364 (3)	N3—C17	1.360 (3)
N2—C8	1.313 (3)	N4—C23	1.326 (3)
N2—C7	1.371 (3)	N4—C22	1.367 (3)
C1—C8	1.453 (3)	C16—C23	1.439 (3)
C1—C9	1.491 (3)	C16—C24	1.495 (3)
C2—C7	1.411 (3)	C17—C22	1.412 (3)
C2—C3	1.418 (3)	C17—C18	1.413 (3)
C3—C4	1.365 (3)	C18—C19	1.369 (3)
C3—H3	0.9500	C18—H18	0.9500
C4—C5	1.422 (3)	C19—C20	1.426 (3)
C5—C6	1.373 (3)	C20—C21	1.367 (3)
C6—C7	1.413 (3)	C21—C22	1.419 (3)
C6—H6	0.9500	C21—H21	0.9500
C8—C15	1.503 (3)	C23—C30	1.500 (3)
C9—C14	1.396 (3)	C24—C29	1.396 (3)
C9—C10	1.398 (3)	C24—C25	1.398 (3)
C10—C11	1.385 (3)	C25—C26	1.392 (3)
C10—H10	0.9500	C25—H25	0.9500
C11—C12	1.392 (4)	C26—C27	1.388 (3)
C11—H11	0.9500	C26—H26	0.9500
C12—C13	1.386 (4)	C27—C28	1.389 (3)
C12—H12	0.9500	C27—H27	0.9500
C13—C14	1.388 (3)	C28—C29	1.394 (3)
C13—H13	0.9500	C28—H28	0.9500
C14—H14	0.9500	C29—H29	0.9500
C15—H15A	0.9800	C30—H30A	0.9800
C15—H15B	0.9800	C30—H30B	0.9800
C15—H15C	0.9800	C30—H30C	0.9800
			
C1—N1—C2	117.45 (18)	C16—N3—C17	118.01 (19)
C8—N2—C7	117.97 (19)	C23—N4—C22	118.16 (19)
N1—C1—C8	121.4 (2)	N3—C16—C23	121.41 (19)
N1—C1—C9	114.73 (19)	N3—C16—C24	115.12 (19)
C8—C1—C9	123.8 (2)	C23—C16—C24	123.47 (19)
N1—C2—C7	121.3 (2)	N3—C17—C22	120.86 (19)
N1—C2—C3	119.08 (19)	N3—C17—C18	119.2 (2)
C7—C2—C3	119.7 (2)	C22—C17—C18	119.91 (19)
C4—C3—C2	120.05 (19)	C19—C18—C17	119.9 (2)
C4—C3—H3	120.0	C19—C18—H18	120.1
C2—C3—H3	120.0	C17—C18—H18	120.1
C3—C4—C5	120.1 (2)	C18—C19—C20	120.4 (2)
C3—C4—Br1	119.14 (16)	C18—C19—Br3	118.16 (17)
C5—C4—Br1	120.71 (17)	C20—C19—Br3	121.42 (16)
C6—C5—C4	120.8 (2)	C21—C20—C19	120.49 (19)
C6—C5—Br2	118.79 (16)	C21—C20—Br4	119.46 (17)
C4—C5—Br2	120.38 (17)	C19—C20—Br4	120.05 (16)
C5—C6—C7	119.6 (2)	C20—C21—C22	120.0 (2)
C5—C6—H6	120.2	C20—C21—H21	120.0
C7—C6—H6	120.2	C22—C21—H21	120.0
N2—C7—C2	120.7 (2)	N4—C22—C17	120.51 (19)
N2—C7—C6	119.68 (19)	N4—C22—C21	120.1 (2)
C2—C7—C6	119.6 (2)	C17—C22—C21	119.3 (2)
N2—C8—C1	121.0 (2)	N4—C23—C16	120.68 (19)
N2—C8—C15	116.12 (19)	N4—C23—C30	115.88 (19)
C1—C8—C15	122.9 (2)	C16—C23—C30	123.34 (19)
C14—C9—C10	119.0 (2)	C29—C24—C25	119.3 (2)
C14—C9—C1	123.8 (2)	C29—C24—C16	122.4 (2)
C10—C9—C1	117.2 (2)	C25—C24—C16	118.25 (19)
C11—C10—C9	120.6 (2)	C26—C25—C24	120.4 (2)
C11—C10—H10	119.7	C26—C25—H25	119.8
C9—C10—H10	119.7	C24—C25—H25	119.8
C10—C11—C12	120.0 (2)	C27—C26—C25	120.0 (2)
C10—C11—H11	120.0	C27—C26—H26	120.0
C12—C11—H11	120.0	C25—C26—H26	120.0
C13—C12—C11	119.8 (2)	C26—C27—C28	119.9 (2)
C13—C12—H12	120.1	C26—C27—H27	120.0
C11—C12—H12	120.1	C28—C27—H27	120.0
C12—C13—C14	120.3 (2)	C27—C28—C29	120.3 (2)
C12—C13—H13	119.8	C27—C28—H28	119.9
C14—C13—H13	119.8	C29—C28—H28	119.9
C13—C14—C9	120.3 (2)	C28—C29—C24	120.1 (2)
C13—C14—H14	119.9	C28—C29—H29	120.0
C9—C14—H14	119.9	C24—C29—H29	120.0
C8—C15—H15A	109.5	C23—C30—H30A	109.5
C8—C15—H15B	109.5	C23—C30—H30B	109.5
H15A—C15—H15B	109.5	H30A—C30—H30B	109.5
C8—C15—H15C	109.5	C23—C30—H30C	109.5
H15A—C15—H15C	109.5	H30A—C30—H30C	109.5
H15B—C15—H15C	109.5	H30B—C30—H30C	109.5
			
C2—N1—C1—C8	1.7 (3)	C17—N3—C16—C23	4.2 (3)
C2—N1—C1—C9	−174.23 (19)	C17—N3—C16—C24	−175.61 (19)
C1—N1—C2—C7	2.7 (3)	C16—N3—C17—C22	1.2 (3)
C1—N1—C2—C3	−177.9 (2)	C16—N3—C17—C18	178.5 (2)
N1—C2—C3—C4	−177.5 (2)	N3—C17—C18—C19	−176.5 (2)
C7—C2—C3—C4	1.8 (3)	C22—C17—C18—C19	0.7 (3)
C2—C3—C4—C5	1.0 (3)	C17—C18—C19—C20	−0.8 (3)
C2—C3—C4—Br1	−177.87 (16)	C17—C18—C19—Br3	178.43 (17)
C3—C4—C5—C6	−2.5 (3)	C18—C19—C20—C21	0.4 (3)
Br1—C4—C5—C6	176.45 (17)	Br3—C19—C20—C21	−178.83 (17)
C3—C4—C5—Br2	176.60 (17)	C18—C19—C20—Br4	−179.61 (17)
Br1—C4—C5—Br2	−4.5 (3)	Br3—C19—C20—Br4	1.2 (3)
C4—C5—C6—C7	0.9 (3)	C19—C20—C21—C22	0.1 (3)
Br2—C5—C6—C7	−178.19 (17)	Br4—C20—C21—C22	−179.92 (16)
C8—N2—C7—C2	0.5 (3)	C23—N4—C22—C17	3.5 (3)
C8—N2—C7—C6	−179.5 (2)	C23—N4—C22—C21	−178.8 (2)
N1—C2—C7—N2	−4.1 (3)	N3—C17—C22—N4	−5.3 (3)
C3—C2—C7—N2	176.6 (2)	C18—C17—C22—N4	177.4 (2)
N1—C2—C7—C6	175.9 (2)	N3—C17—C22—C21	177.0 (2)
C3—C2—C7—C6	−3.4 (3)	C18—C17—C22—C21	−0.3 (3)
C5—C6—C7—N2	−177.9 (2)	C20—C21—C22—N4	−177.9 (2)
C5—C6—C7—C2	2.0 (3)	C20—C21—C22—C17	−0.2 (3)
C7—N2—C8—C1	3.9 (3)	C22—N4—C23—C16	1.9 (3)
C7—N2—C8—C15	−176.87 (19)	C22—N4—C23—C30	−174.46 (19)
N1—C1—C8—N2	−5.3 (3)	N3—C16—C23—N4	−6.1 (3)
C9—C1—C8—N2	170.3 (2)	C24—C16—C23—N4	173.75 (19)
N1—C1—C8—C15	175.5 (2)	N3—C16—C23—C30	170.0 (2)
C9—C1—C8—C15	−8.9 (3)	C24—C16—C23—C30	−10.1 (3)
N1—C1—C9—C14	−134.7 (2)	N3—C16—C24—C29	138.6 (2)
C8—C1—C9—C14	49.4 (3)	C23—C16—C24—C29	−41.3 (3)
N1—C1—C9—C10	43.5 (3)	N3—C16—C24—C25	−37.6 (3)
C8—C1—C9—C10	−132.4 (2)	C23—C16—C24—C25	142.5 (2)
C14—C9—C10—C11	0.4 (3)	C29—C24—C25—C26	1.5 (3)
C1—C9—C10—C11	−177.8 (2)	C16—C24—C25—C26	177.9 (2)
C9—C10—C11—C12	0.6 (4)	C24—C25—C26—C27	−0.6 (3)
C10—C11—C12—C13	−0.9 (4)	C25—C26—C27—C28	−1.0 (3)
C11—C12—C13—C14	0.3 (4)	C26—C27—C28—C29	1.7 (4)
C12—C13—C14—C9	0.7 (4)	C27—C28—C29—C24	−0.7 (3)
C10—C9—C14—C13	−1.1 (3)	C25—C24—C29—C28	−0.9 (3)
C1—C9—C14—C13	177.1 (2)	C16—C24—C29—C28	−177.1 (2)

### 6,7-Dibromo-2-methyl-3-phenylquinoxaline Hydrogen-bond geometry (Å, º)

**Table:**

*D*—H···*A*	*D*—H	H···*A*	*D*···*A*	*D*—H···*A*
C6—H6···Br3^i^	0.95	2.91	3.784 (2)	154
C6—H6···Br4^i^	0.95	3.09	3.834 (2)	136
C15—H15*B*···Br1^ii^	0.98	3.13	4.044 (2)	155
C21—H21···Br1	0.95	3.05	3.741 (2)	131
C13—H13···N3^iii^	0.95	2.62	3.495 (3)	152
C30—H30*B*···N3^iv^	0.98	2.87	3.231 (3)	103
C28—H28···N1^v^	0.95	2.68	3.568 (3)	156

Symmetry codes: (i) −*x*, *y*+1/2, −*z*+1/2; (ii) *x*, −*y*+3/2, *z*−1/2; (iii) *x*, *y*+1, *z*; (iv) *x*, −*y*+1/2, *z*+1/2; (v) −*x*+1, *y*−1/2, −*z*+3/2.
